# Hsp40 Protein LeDnaJ07 Enhances the Thermotolerance of *Lentinula edodes* and Regulates IAA Biosynthesis by Interacting LetrpE

**DOI:** 10.3389/fmicb.2020.00707

**Published:** 2020-04-17

**Authors:** Gangzheng Wang, Yi Luo, Chen Wang, Yan Zhou, Chunye Mou, Heng Kang, Yang Xiao, Yinbing Bian, Yu Hua Gong

**Affiliations:** ^1^Institute of Applied Mycology, College of Plant Science and Technology, Huazhong Agricultural University, Wuhan, China; ^2^State Key Laboratory of Applied Microbiology Southern China, Guangdong Provincial Key Laboratory of Microbial Culture Collection and Application, Guangdong Open Laboratory of Applied Microbiology, Guangdong Institute of Microbiology, Guangzhou, China

**Keywords:** DnaJ protein, *Lentinula edodes*, thermotolerance, IAA, yeast two-hybrid, BiFC, LetrpE

## Abstract

Our previous study found that *LeDnaJ07* RNAi decreased *Lentinula edodes* resistance to heat stress and *Trichoderma atroviride* infection. In this study, the structure and function of the *LeDnaJ07* gene was analyzed by gene cloning and overexpression in *L. edodes* stress-sensitive strain YS55 via the *Agrobacterium*-mediated transformation method. Transformants were confirmed by qRT-PCR, fluorescence observation and Southern blotting. Overexpression of *LeDnaJ07* in YS55 not only enhanced *L. edodes* mycelial resistance to heat stress but also facilitated mycelial growth. In the presence of heat stress, the intracellular IAA content showed a significant increase in the two *LeDnaJ07* overexpression strains but only a slight change in the YS55 wild type strain. Moreover, the interaction between LeDnaJ07 and LetrpE was demonstrated via Y2H and BiFC assays. These results suggested that LeDnaJ07 may be involved in regulating IAA biosynthesis and the resistance of *L. edodes* to heat stresses via interacting with LetrpE.

## Introduction

Heat stress is a major constraint in mushroom production. High temperature not only inhibits mycelial growth and fruiting body formation, but also enhances *Trichoderma* sp. infection and cell membrane fluidity in edible fungi (Lu et al., 2014; Liu et al., 2017; Qiu et al., 2017). Previous studies have reported that Para-aminobenzoic acid (PABA) synthase and nitric oxide reduced ROS (reactive oxygen species) accumulation to alleviate oxidative damage induced by heat stress (Kong et al., 2012a; Lu et al., 2014); catalase, trehalose, HSP and calcium-calmodulin play an important role in regulating the response of the edible fungi to heat stress (Kong et al., 2012b; Deng et al., 2014; Wang et al., 2017; Liu et al., 2018).

*Lentinula edodes*, one mushroom used for decoctions and essences and alternative medicine, is widely cultivated in the world and ranks second in worldwide production next to *Agaricus bisporus* (Chang, 2005; Finimundy et al., 2014). During mycelial culture of *L. edodes* in rotted logs or bags, six *Trichoderma* species have been isolated and identified in previous studies, and these studies reported that high temperatures caused large losses in *L. edodes* yield (Cao et al., 2015; Wang G.Z. et al., 2016). Our previous studies found that *LeDnaJ07* (named as *LeDnaJ* in our previous reports) and auxin participated in the response to heat stress in *L. edodes* (Wang et al., 2018a, b; Zhou et al., 2018).

Heat shock protein 40 (termed DnaJ or J protein), an important member of the heat shock protein family, plays a critical role in modulating the growth and development processes of organism as well as their resistance to abiotic and biotic stresses (Caplan et al., 1992; Suetsugu et al., 2005; Shen and Yu, 2011; Zhou et al., 2012). In plants, the function of DnaJ protein is well-known. DnaJ proteins regulate the resistance of plants to abiotic stresses (heat, cold, salt, and drought) via inducing cell elongation and stabilizing the structure of the photosynthetic system II (Bekhochir et al., 2013; Kong et al., 2014b; Wang et al., 2019). In addition, overexpression of DnaJ protein enhanced the resistance of tobacco to *Phytophthora parasitica* pv nicotianae and *Sclerotinia sclerotiorum* via regulating the activity of ascorbic acid peroxidase (APX), Mn-SOD (superoxide) and HSP70 (Rampuria et al., 2018). Moreover, the knockdown of OsDjA6 enhanced the resistance of *Oryza sativa* to *Magnaporthe oryzae* (Zhong et al., 2018). For *Candida albicans*, Ydj1 loss resulted in the failure of morphogenetic switch from yeast to filamentous form (Xie et al., 2017). However, the function of DnaJ protein in macro basidiomycete fungi is less well-known.

Based on our previous studies, the transcript and protein levels of LeDnaJ07 were dramatically upregulated in the heat-resistant strain S606 after heat stress. *LeDnaJ07* overexpression in *L. edodes* strain S606 enhanced the thermotolerance via regulating IAA biosynthesis, while *LeDnaJ07* RNAi in *L. edodes* strain S606 played a negative role in regulating mycelial growth, the resistance to heat stress and *T. atroviride* and IAA biosynthesis after heat stress (Wang et al., 2018a, b). According to transcriptome and proteome analyses after heat stress, the protein level of LetrpE, a rate-limiting enzyme in the IAA biosynthesis pathway, in the thermotolerance strain S606 was increased by approximately 500-fold after heat stress, while its transcript level was obviously downregulated (Wang et al., 2018a). Further, *LetrpE* RNAi in S606 played a negative role in regulating *L. edodes* thermotolerance (Ma et al., 2018).

The objectives of the present study were as follows: (1) to analyze the structure of *LeDnaJ07* in *L. edodes* thermotolerance strain S606 and stress-sensitive strain YS55; (2) to explore the effect of *LeDnaJ07* on mycelial growth and resistance to heat stress in YS55; (3) to quantify the IAA content after 24 h of 38°C heat stress in sawdust medium; and (4) to verify the interaction between LeDnaJ07 and LetrpE via Y2H (yeast two-hybrid) and BiFC (bimolecular fluorescence complementation) analyses.

## Materials and Methods

### Strains and Culture Conditions

*L. edodes* strain YS55 was used as the recipient host strain for gene overexpression and it was cultured at 25°C on MYG (malt yeast glucose) medium or sawdust medium. *E. coli* Trans1-T1 and *Agrobacterium tumefaciens* EHA105 were used for plasmid amplification and infecting *L. edodes* mycelia, respectively, and they were cultured in 50 μg/ml kanamycin Luria–Bertani (LB) medium.

### Extraction of RNA, LeDnaJ07 Cloning and Sequence Analysis

Total RNA was extracted from *L. edodes* strain S606 and YS55 using the RNAiso Plus (TAKARA, Shanghai, China) method, and isolated RNA was then reverse-transcribed into cDNA using the HiScript II One Step RT-PCR Kit (Vazyme, Nanjing, China) according to the manufacturer’s instruction. Based on *L. eodes* strain WX-1 genome information^1^ (Chen et al., 2016), the CDS (coding sequence) of LeDnaJ07 (Le01Gene01273) was amplified by PCR using *L. edodes* strain S606 and YS55 cDNA (primers listed in Supplementary Table S1), and the fragments were cloned into the pEASY-Blunt vector (TransGen Biotech, Beijing, China) for sequencing. The CDSs containing S606 and YS55 as well as WX-1 were aligned using ClustalW in MEGA 6.0, and the alignment result was visualized by GeneDoc. The protein sequences of *LeDnaJ07* were aligned with 22 J proteins in *Saccharomyces cerevisiae* using the neighbor-joining method in MEGA 6.0.

### Vector Construction and Fungal Transformation

The fungal overexpression vector was constructed as previously described (Ding et al., 2011). The *Legpd* (*L. edodes* glyceraldehyde-3-phosphate dehydrogenase) promoter was used to induce expression of hygromycin phosphotransferase, and the *Leactin* promotor was used to induce the mCherry fluorescent protein (mRFP) and *LeDnaJ07*. The coding sequences of mRFP and LeDnaJ07 were obtained by PCR using primers with the homologous arms containing restriction enzyme sites (*Eco*R**I** and *Kpn***I**) as well as pBlueScript SK plasmid and *L. edodes* cDNA as the template (Supplementary Table S1). The overexpression vector and control vector only containing mCherry protein were transferred into the *L. edodes* strain by *A. tumefaciens* infection. Nine transformants were selected randomly to analyze the overexpression efficiency of the transformants by qRT-PCR. mRFP expression was assessed by observing fluorescence using a fluorescence microscope, and the stable integration of the target gene into the genome of *L. edodes* was confirmed by Southern blot analysis (Yin et al., 2015). Two independent overexpression strains with the highest upregulation efficiency and one transformant (control strain) only containing mCherry protein were selected for further study.

### Mycelial Growth and Thermotolerance Susceptibility Assays

After 10-day growth at 25°C, the colony diameters of the transgenic and WT strains in the MYG and sawdust medium were measured to evaluate the effect of *LeDnaJ07* on *L. edodes* mycelial growth. Moreover, the *LeDnaJ07* over-expressed, control and WT strains were grown in plastic culture bags (15 cm in fold diameter and 30 cm in length) containing 0.8 kg (wet weight) of sterilized sawdust medium (78% hardwood, 20% wheat bran, 1% lime, 1% gypsum and 55% water content). Five mycelial plugs (8 mm in diameter) were inoculated into each culture bag, and 12 culture bags were prepared separately for each of the transformant and wild-type (WT) strains. Culture bags were followed by culture at 25°C in the dark.

To test the effect of *LeDnaJ07* overexpression on the resistance of *L. edodes* against heat stress, thermotolerance assays, involving short- and long- term heat stresses, were performed according to our previous method (Wang et al., 2018b). In the short-term heat stress group, the stress temperature and recovery time were 38°C and 25 d, respectively. Colony diameters and characteristics were used to quantify the sensitivity of the transgenic and WT strains to heat stress, with five replicates for each group.

### Detection and Measurement of IAA

*L. edodes* mycelia treated or untreated by 38°C heat stress for 24 h were collected separately. To analyze IAA differences between transformant and WT strains, mycelial samples were grinded into fine powder in liquid nitrogen. IAA extraction and measurement methods were performed according to our previous study (Wang et al., 2018b).

### Yeast Two-Hybrid and Bimolecular Fluorescence Complementation Analyses

The interaction between LeDnaJ07 with LetrpE was verified using the Matchmaker Gold Yeast Two-Hybrid (Y2H) System (Clontech, Palo Alto, CA, United States) (Yin et al., 2015). LeDnaJ07 and LetrpE were introduced into pDEST22 and pDEST32 plasmids as baits and preys, respectively. The bait and prey plasmids were co-transformed into *S. cerevisiae* strains MaV103 and MaV203 according to the manufacturer’s instructions. Transformed yeast cells were assayed for growth onsynthetic dropout SD/-Trp-Leu-Ura plates, SD/-Trp-Leu-His plates and SD/-Trp-Leu plates containing X-a-galactosidase (X-a-gal) or 3-amino-1,2,4-triazole (3AT) (10 and 25 mM).

The full-length cDNA sequences of LeDnaJ07 and LetrpE were amplified by PCR and cloned into the *Bam*HI/*Xho*I site of pSYCE-MR and pSCYNE-R vectors, respectively, generating CFPC-LeDnaJ07/LetrpE and LeDnaJ07/LetrpE-CFPN fusion constructs. The constructs were transformed into *A. tumefaciens* strain AGL-1. Transient expression of proteins in *N. benthamiana* leaves by *A. tumefaciens* infiltration was conducted as previously described (Kang et al., 2011; Yang et al., 2018). Cyan fluorescence of fusion proteins was assayed 2–3 d after infiltration using a Zeiss LSM510 with excitation/emission wavelengths of 405 and 477 nm, respectively.

### Statistical Analysis

All data averaged from the five independent sample measurements were used to ensure the reliability and reproducibility of the change trend between WT and overexpression strains. The significance of the differences between the analyzed samples was determined by one-way analysis of variance (ANOVA) and tested for significant (*P* < 0.05) treatment differences using Duncan’s multiple range test.

## Results

### Analysis of LeDnaJ07 CDS in Different *L. edodes* Strains

**FIGURE 1:**
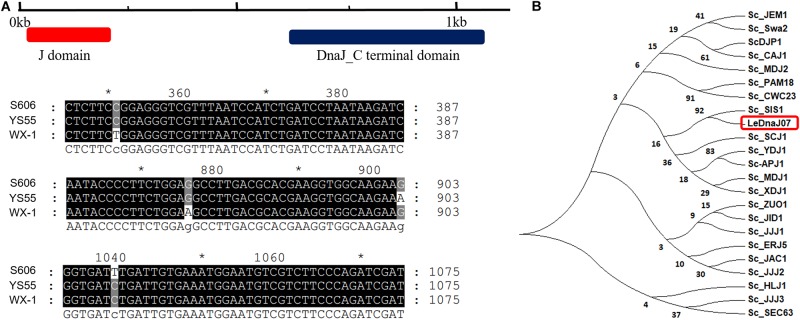
*LeDnaJ07* sequence analysis. **(A)** Structure analysis and sequence alignment of *LeDnaJ07* CDS in three *L. edodes* strains. **(B)** Molecular phylogenetic tree of *LeDnaJ07* generated with the neighbor-joining (NJ) method using MEGA 7.0. An unrooted phylogenetic tree was generated based on the alignment of the amino acid (AA) sequences containing LeDnaJ07 and 22 *S. cerevisiae* J proteins.

The CDS difference of *LeDnaJ07* between heat-resistant strain S606, stress-sensitive strain YS55 and cultivation strain WX-1 was analyzed using the PCR sequecing result. According to the prediction of DnaJ domain in https://www.ncbi.nlm.nih.gov/Structure/cdd/wrpsb.cgi, J domain located from 10 to 198 bp in *LeDnaJ07* CDS (Figure 1A). In terms of sequence alignment, it was found that only four of 1119 base pairs out of J domain exhibited a difference among three *L. edodes* strains (Figure 1A and Supplementary Figure S1), which made no changes on the amino acid sequence of three LeDnaJ07 proteins. This result indicated that *LeDnaJ07* proteins in different *L. edodes* strains were identical. As shown in Figure 1B, *LeDnaJ07* exhibited a 92% similarity to *SIS1* in *S. cerevisiae*, indicating that *LeDnaJ07* could play an important role during translation initiation.

### Construction of LeDnaJ07 Overexpression Strains

**FIGURE 2:**
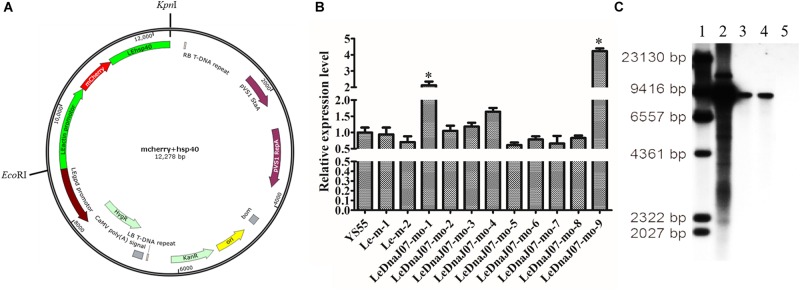
Vector map and identification of *LeDnaJ07* overexpression transformants. **(A)** Vector map of *LeDnaJ07* overexpression. **(B)** qRT-PCR analysis of *LeDnaJ07* overexpression transformants. YS55, wild type strain; Le-m-1/2, Control transgenic strains; LeDnaJ07-mo-1-9, *LeDnaJ07* overexpression strains; *, represents the significant difference. **(C)** Southern blot analysis of genomic DNA isolated from the transformants and WT strain digested with *Hin*dIII. Lane 1-5, Maker, pCAMBIA1300-g plasmid, LeDnaJ07-mo-1, LeDnaJ07-mo-9, and the WT strain YS55, respectively.

**FIGURE 3:**
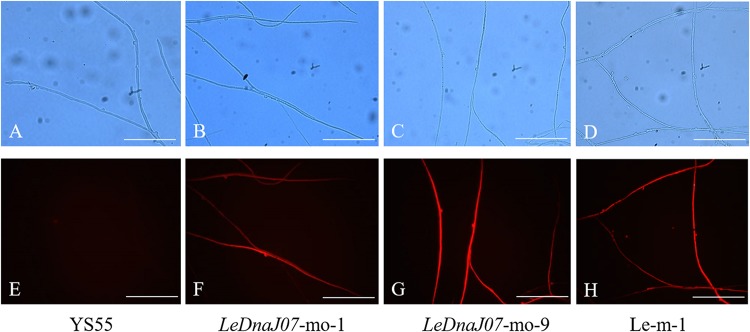
Expression of mCherry in the *LeDnaJ07* overexpression transformant, control transgenic and WT strains. **(A–D)** Detection of *L. edodes* mycelia without fluorescence. **(E–H)** Detection of *L. edodes* mycelia with fluorescence under red light. Bar, 10 μm.

The overexpression vector pCAMBIA1300-g-mo was used to express the fusion protein mRFP-*LeDnaJ07*, and the hygromycin B resistance gene in that overexpression vector was treated as the selectable marker (Figure 2A). Nevertheless, the vector pCAMBIA1300-g-m only expressing the mRFP was treated the contorl vector. After introducing the expression vector and the control vector into the mycelia of *L. edodes* via *A. tumefaciens* mediation, the expression level of *LeDnaJ07* was used to confrim the transformants. Among all the transformants, two overexpression transformants LeDnaJ07-mo-1 and LeDnaJ07-mo-9 had an about two and four-fold upregulation (*P* < 0.05), respectively, but no significant expression changes found in other transformatns and two control transformants Le-m-1 and Le-m-2 (Figure 2B). Southern blot showed that one copy of the insert fragment was found in overexpression strains LeDnaJ07-mo-1 and LeDnaJ07-mo-9, but no copy in the WT strain (Figure 2C), indicating the integration of the insert fragment including in *LeDnaJ07* gene into *L. edodes* genome. To verify the expression level of the introduced mCherry reporter gene, mycelia fluorescence of three individual transformants grown on MYG medium were detected using a fluorescence microscope. As shown in Figure 3, the clear distribution of red fluorescence was found in the mycelia of three individual transformants. These results suggested that the *LeDnaJ07* overexpression recombinants were successfully obtained.

### Overexpression of LeDnaJ07 Enhances the Mycelial Growth of *L. edodes*

**FIGURE 4:**
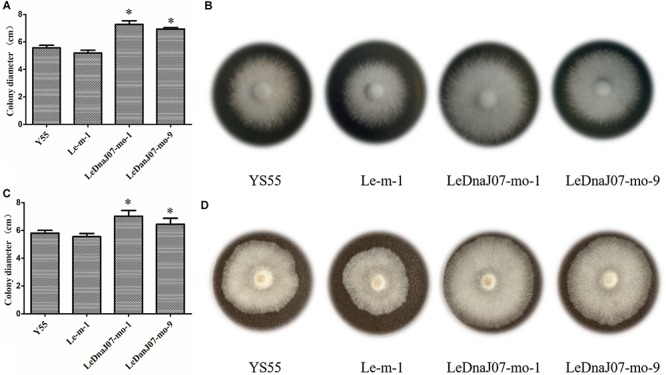
Effect of *LeDnaJ07* overexpression on the growth of *L. edodes* mycelia. **(A)** and **(B)** Colony characteristics and diameters of the *LeDnaJ07* overexpression transformant, control transgenic and the WT strains in MYG medium after 9 days of inoculation. **(C)** and **(D)** Colony characteristics and diameters of the *LeDnaJ07* overexpression transformant, control transgenic and WT strains in sawdust medium after 15 days of inoculation. Each bar represents mean ± standard deviation (SD) (*n* = 5). *, The colony diameters of the *LeDnaJ07* overexpression transformants were significantly different from those of the WT and control transgenic strains (*P* < 0.05).

As shown in Figure 4, in MYG medium, the colony diameters of the two overexpression transformants (LeDnaJ07-mo-1 and LeDnaJ07-mo-9) were 7.28 and 6.93 cm after 9 days of inoculation, with 5.57 and 5.20 cm diameters for the control and WT strains (Figures 4A,B); and the colony diameters of the two overexpression transformants were 7.03 and 6.45 cm after inoculation for 15 days in sawdust medium, with diameters of 5.80 and 5.55 cm for the control and WT strain (Figures 4C,D). Besides, it was documented that the mycelia of *LeDnaJ07* over-expressed transformants were full of the cultivation bags after 45 days of growth, while the mycelia of WT strain YS55 and control strain Le-m-1 covered 70% of the cultivation bags (not shown), indicating that improving the expression of *LeDnaJ07* could promote the growth of *L. edodes* mycelia in MYG and sawdust medium.

### LeDnaJ07 Overexpression Is of Benefit to *L. edodes* Thermotolerance

**FIGURE 5:**
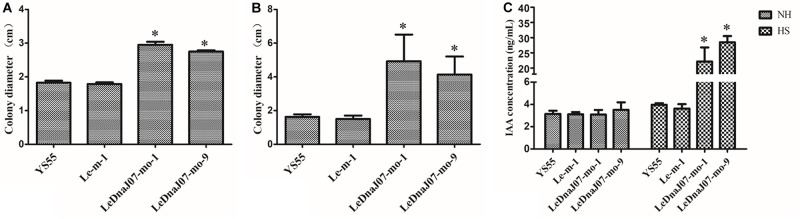
*LeDnaJ07* overexpression enhances *L. edodes* thermotolerance and IAA biosynthesis after heat stress. **(A)** Colony diameters of the WT, control transgenic and *LeDnaJ07* overexpression transformant strains grown in sawdust medium for 15 days of recovery from the 15 days of 31°C heat stress. **(B)** Colony diameters of the WT, control transgenic and *LeDnaJ07* overexpression transformant strains in sawdust medium after 25 days of recovery from the 24 h of 38°C heat stress. **(C)** IAA concentration change in the wild type YS55 and *LeDnaJ07* overexpression transformants after 38°C heat stress for 24 h. Each bar represents mean ± standard deviation (SD) (*n* = 5). NH, not heat stress. HS, heat stress. *, significant difference (*P* < 0.05).

The effect of *LeDnaJ07* on *L. edodes* thermotolerance was estimated by comparing the colony diameters after heat stress among WT, control and *LeDnaJ07* overexpression transformant strains. Long-term heat stress lasted for 15 days at 31°C, and short-term heat stress lasted 24 h at 38°C. From the perspective of the thermotolerance assays, no growth was observed in the mycelia of all strains after 31°C heat stress for 15 days. Nevertheless, after 15 days of recovery at 25°C, only the mycelia of the two *LeDnaJ07* overexpressed transformants with a colony diameter about 3 cm regrew at 31°C heat stress, whereas an about 2 cm colony diameter was found in WT and control transgenic strains (Figure 5A). After 24 h of heat stress at 38°C, the mycelia of the two overexpression transformants showed a colony diameter about 4.5 cm after a 25-day recovery at 25°C, but the mycelia of control transgenic and WT strains showed a colony diameter of 2.0 cm and were dead and dissolved (Figure 5B). The similar results of the long-term and short-term heat stress assays demonstrated that LeDnaJ07 promoted the recovery capacity and thermotolerance of *L. edodes* mycelia in response to heat stress.

### The Overexpression of *LeDnaJ07* Promotes Intracellular IAA Biosynthesis After Heat Stresses

At 25°C, the intracellular IAA concentrations of YS55, Le-m-1 and the two *LeDnaJ07* overexpression strains displayed a slight difference, but they showed significant differences after heat stress (Figure 5C). After 24 h of heat stress at 38°C, the intracellular IAA concentration had a 7.16 and 8.12-fold increase in the two LeDnaJ07 overexpression transformants, but only a 1.26 and 1.17-fold increase in the WT strain YS55 and control transgenic strain Le-m-1, indicating that LeDnaJ07 was beneficial to IAA biosynthesis during the response to heat stress.

### Interaction Between LeDnaJ07and LetrpE in Yeast and Tobacco Leaf Cells

**FIGURE 6:**
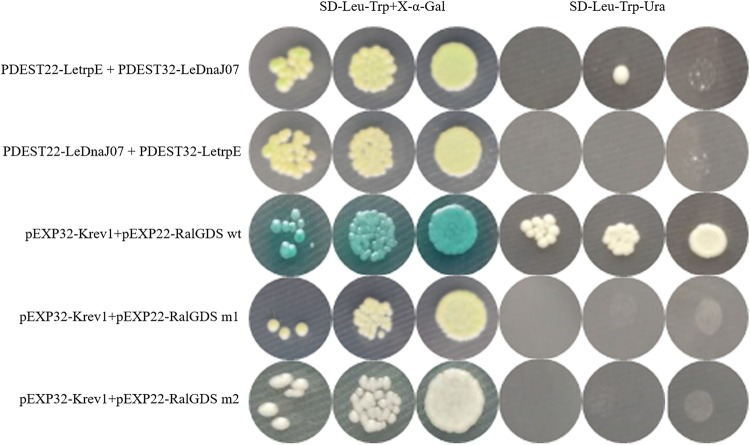
Interaction verification of LeDnaJ07 with LetrpE in the Y2H system on the plates with SD/-Leu-Trp and SD/-Leu-Trp-Ura. Tested groups, DEST22-LeDnaJ07 + PDEST32-LetrpE, and PDEST22-LetrpE + PDEST32-LeDnaJ07; Positive control, pEXP32-Krev1 + pEXP22-RalGDS wt; Weak position interaction control, pEXP32-Krev1 + pEXP22-RalGDS m1; Negative control, pEXP32-Krev1 + pEXP22-RalGDS m2.

**FIGURE 7:**
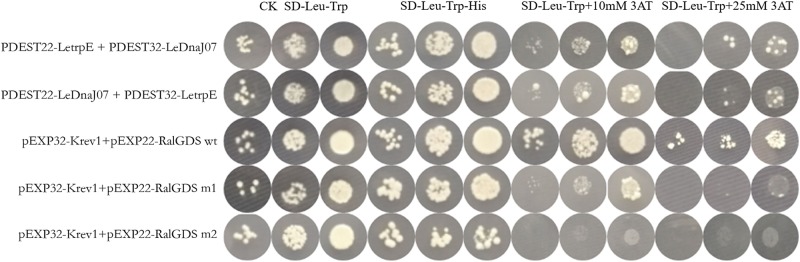
Interaction verification of LeDnaJ07 with LetrpE in the Y2H system on the plates with SD/-Leu-Trp-His and SD/-Leu-Trp and different concentration 3AT. Tested groups, DEST22-LeDnaJ07 + PDEST32-LetrpE and PDEST22-LetrpE + PDEST32-LeDnaJ07; Positive control, pEXP32-Krev1 + pEXP22-RalGDS wt; Weak position interaction control, pEXP32-Krev1 + pEXP22-RalGDS m1; Negative control, pEXP32-Krev1 + pEXP22-RalGDS m2.

**FIGURE 8:**
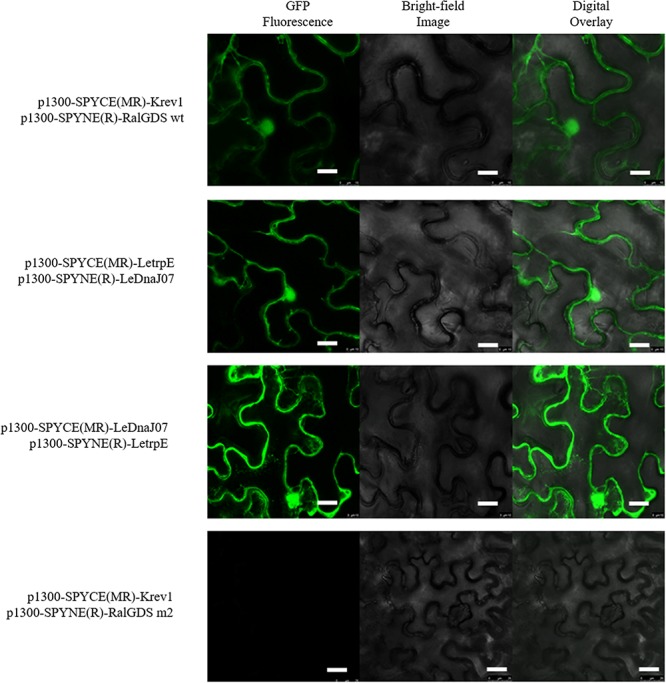
Interaction of LetrpE with LeDnaJ07 in plant cells. Tested groups, p1300-SPYCE(MR)-LetrpE + p1300-SPYNE(R)-LeDnaJ07 and p1300-SPYCE(MR)-LeDnaJ07 + p1300-SPYNE(R)-LetrpE, Positive control, p1300-SPYCE(MR)-Krev1 + p1300-SPYNE(R)-RalGDS wt; Negative control, p1300-SPYCE(MR)-Krev1 + p1300-SPYNE(R)-RalGDS m2(negative control). Bar, 10 μm.

According to the result of self-activation assay, it was found that the yeast cells only containg one of LeDnaJ07 and LetrpE could grow ont SD/-Trp plates, but no growth found on SD/-Trp-Leu and SD/-Trp-Ade plates, suggesting that they showed no self-activation effect in transgenic yeast cells (Supplementary Figure S2). In the Y2H assay, after 3 days of culture at 30°C, yeasts from all mixtures grew on SD/-Trp-Leu plates (Figure 6). The yeasts from mixture containing the positive control (pEXP32-Krev1 + pEXP22-RalGDS wt) turned blue, and the yeasts from the weak positive interaction control (pEXP32-Krev1 + pEXP22-RalGDS m1) and the tested group (PDEST22-LeDnaJ07 + PDEST32-LetrpE and PDEST22-LetrpE + PDEST32-LeDnaJ07) exhibited light blue. Whereas, the yeasts from the negative control (pEXP32-Krev1 + pEXP22-RalGDS m2) displayed white. In addition, the mated yeasts from the positive control and tested groups exhibited the growth on SD/-Trp-Leu-Ura plates (Figure 6). Except for the negative control, the mated yeasts of other four groups grew on SD/-Leu-Trp plates with 10 and 25 mM 3AT (Figure 7). The above results indicated that a weak interaction occurred between LeDnaJ07 and LetrpE. Furthermore, as shown in Figure 8, the BiFC assay showed that cells from the positive control (p1300-SPYCE(MR)-Krev1 + p1300-SPYNE(R)-RalGDS wt) and tested groups (p1300-SPYCE(MR)-LeTrpE + p1300-SPYNE(R)-LeDnaJ07 and p1300-SPYNE(R)-LetrpE + p1300-SPYCE(MR)-LeDnaJ07) exhibited green fluorescence, but no green fluorescence was observed in the negative group (p1300-SPYCE(MR)-Krev1 + p1300-SPYNE(R)-RalGDS m2). Taken together, these data verified that LeDnaJ07 had an interaction with LetrpE.

## Discussion

Numerous studies have shown that DnaJ proteins are involved in biotic and abiotic stresses for human and plants (Shen and Yu, 2011; Kakkar et al., 2012; Lee et al., 2018; Zhong et al., 2018). However, the regulation of the DnaJs on the resistance of higher basidiomycetes to biotic and abiotic stresses remains poorly understood. According to our previous studies, it was documented that the RNAi of the *LeDnaJ07* decreased mycelial growth and the resistance to heat stress, while *LeDnaJ07* overexpression enhanced the thermotolerance of *L. edodes* heat-resistant strain S606 (Wang G.Z. et al., 2016, Wang et al., 2018b). However, it is worthy of study whether *LeDnaJ07* overexpression regulates the resistance of other *L. edodes* strains to stresses or not, how *LeDnaJ07* regulates the response of *L. edodes* to thermotolerance. In this study, we intended to provide evidence that J domain in different *L. edodes* strains was consistent, and the overexpression of the chaperone *LeDnaJ07* participated in regulating thermotolerance and mycelial growth by targeting LetrpE in the stress-sensitive strain YS55.

In plant, a large number of papers reported that DnaJ proteins regulated growth and development. For *Arabidopsis thaliana* and rice, DnaJ protein loss led to an obvious late-flowering trait and largely abnormal cellular structures (Shen and Yu, 2011; Zhu et al., 2015). Overexpression of *BIL2*, one gene encoding the mitochondrial DnaJ protein, promoted elongation of *A. thaliana* cells by promoting ATP synthesis (Bekhochir et al., 2013). Silencing of *PSA2*, which encodes DnaJ family proteins, led to an obvious decrease in chlorophylls and total carotenoids, further variegated leaves and retarded growth (Wang Y.W. et al., 2016). The present study documented that colony diameters of two overexpression transformants were significantly larger than them of the control transgenic and WT strains on sawdust and MYG medium at the same culture condition (Figure 4), indicating that *LeDnaJ07* overexpression exhibited a positive role in regulating *L. edodes* mycelial growth and development. These results were consistent with the results of our previous study that *LeDnaJ07* silence resulted in a dramatically retarded mycelial growth in the heat-resistant strain S606 (Wang et al., 2018b). Nevertheless, it is still unknown how LeDnaJ07 overexpression promotes mycelial growth.

Furthermore, an increasing number of reports have demonstrated that the high expression and accumulation of DnaJ proteins positively regulated the thermotolerance of plants. For example, the double knock-out of *AtDjA2* and *AtDjA3* impaired the thermotolerance of *A. thaliana* young seedlings, whereas overexpression of both genes increased the thermotolerance of transgenic plants (Li et al., 2007). In transgenic tomatoes, DnaJ protein overexpression facilitated heat tolerance by regulating ROS and H_2_O_2_ and protected Rubisco activity from degradation under heat stress by keeping the levels of proteolytic enzymes low (Kong et al., 2014a; Wang et al., 2015, 2019). In the present study, the mycelia of the two *LeDnaJ07* overexpression strains regrew after 15-day of heat stress at 31°C and 24 h of heat stress at 38°C, but no growth was observed in the control transgenic and WT strains at these conditions (Figures 5A,B). In addition, we observed that mycelia of the control transgenic and WT strains were dissolved after 38°C heat stress, whereas the overexpression strains were mildly affected by heat stress. This difference in the phenotype trait of the test *L. edodes* strains in response to heat stress demonstrated that the *LeDnaJ07* overexpression alleviated heat stress, providing further evidence that DnaJ proteins can enhance the thermotolerance in different *L. edodes* strains.

The present study also showed that overexpression of *LeDnaJ07* in stress-sensitive strain YS55 promoted the IAA biosynthesis under heat stress (Figure 5C), which is consistent with our previous studies. *LeDnaJ07* RNAi and overexpression decreased and promoted, respectively, the intracellular IAA concentration after heat stress, and 0.01 mM IAA partly restored the resistance of *LeDnaJ07* RNAi strains to heat stresses (Wang G.Z. et al., 2016, Wang et al., 2018b), indicating that *LeDnaJ07* is quite important for IAA biosynthesis under heat stress condition. Anthranilate synthase TrpE is a key rate-limiting enzyme in the tryptophan-dependent IAA biosynthesis pathway (Ishimoto et al., 2010). In our study, the yeast two-hybrid system and BiFC assays proved that LeDnaJ07 interacts with *L. edoeds* LetrpE (Figures 6–8), suggesting that the LeDnaJ07/LetrpE machinery possibly may play a significant role in thermotolerance by regulating IAA biosynthesis. According to our previous study, exogenous IAA and its analoges may alleviate the effect of oxidative damage induced by heat stress on the mycelia of *L. edodes* by regulating the expression of redox enzymes, such as SOD and LOX (Zhou et al., 2018). These results demonstrated that LeDnaJ07 overexpression enhanced the extracellular IAA content by interacting with LetrpE (a rate-limiting enzyme in the IAA biosynthesis pathway), and then improved the thermotolerance of *L. edodes* mycelia by mediating redox enzymes.

## Conclusion

**FIGURE 9:**
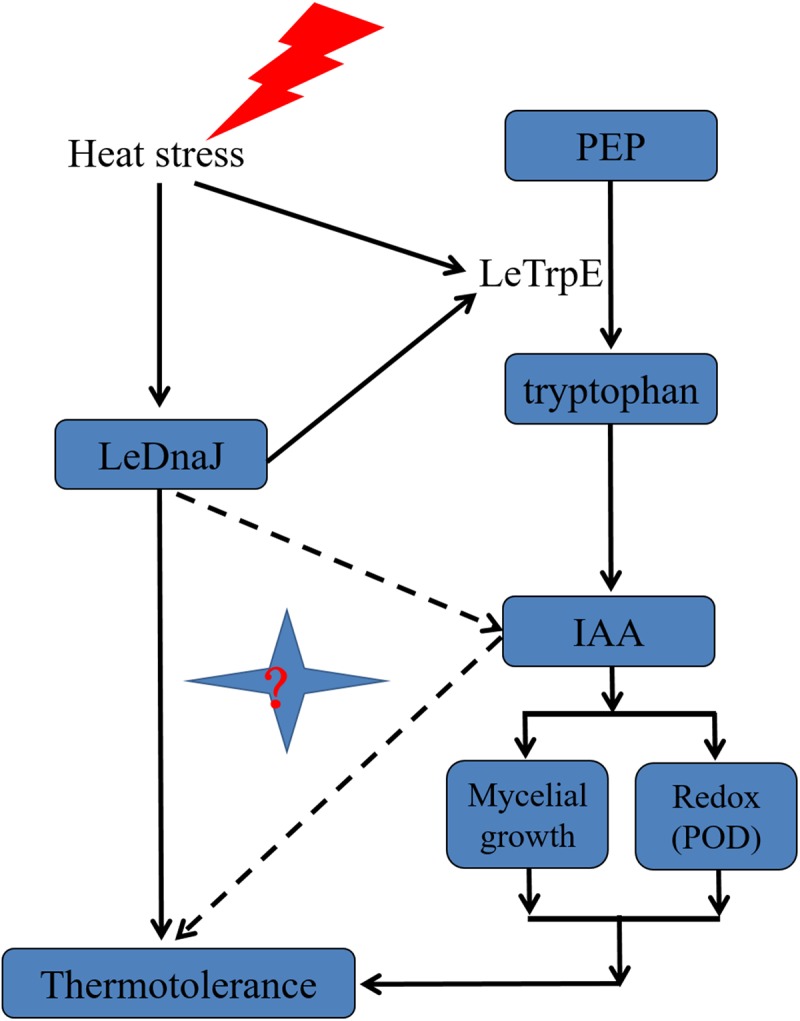
Schematic model for the role of IAA and LeDnaJ07 in enhancing thermotolerance of *L. edodes*.

In summary, we cloned and analyzed the *LeDnaJ07* gene in *L. edodes* heat-resistant strain S606 and stress-sensitive strain YS55. Overexpression of *LeDnaJ07* in YS55 enhanced the tolerance to heat stress in the transgenic strains, with an obvious increase in mycelial growth and intracellular IAA concentration in the transgenic strains compared with the control transgenic and WT strains. In addition, the present study for the first time showed that LeDnaJ07 interacted with LetrpE. We constructed a model for the role of LeDnaJ07 and the IAA signal pathway in enhancing *L. edodes* thermotolerance (Figure 9). This study provides valuable information for the relative expression level of DnaJ proteins in stress-resistant mushroom breeding. Further study will focus on the mechanism by which LeDnaJ07 regulates *L. edodes* thermotolerance via the IAA signal pathway.

## Data Availability Statement

All datasets generated for this study are included in the article/Supplementary Material.

## Author Contributions

GW, YZ, HK, YX, YB, and YG conceptualized the study. CW helped with the data curation. GW, YL, and YG contributed to the formal analysis. YB was responsible for the funding acquisition. YL and CW carried out the investigation. GW, YL, CM, HK, and YG worked on the methodology. YX contributed to the resources. GW, YL, and CW were responsible for the validation. GW, YL, and CM helped with the visualization. GW wrote the original draft. YZ, YB, and YG reviewed and edited the manuscript.

## Conflict of Interest

The authors declare that the research was conducted in the absence of any commercial or financial relationships that could be construed as a potential conflict of interest.

## Footnotes

1 http://legdb.chenlianfu.com/index.html
