# Supplementary material for: Hsp40 Protein LeDnaJ07 Enhances the Thermotolerance of Lentinula edodes and Regulates IAA Biosynthesis by Interacting LetrpE
Source: Front Microbiol. 2020 Apr 17;11:707. doi: 10.3389/fmicb.2020.00707 (PMC7180325; doi:10.3389/fmicb.2020.00707)
Supplement: Supplementary file 1 [file Data_Sheet_1.PDF]

Figure S1 The detailed information of sequence alignment of *LeDnaJ07* CDS in three *L. edodes* strains.

```
                     *        20         *        40
S606 : ATGGGTACTGACTACTACAAGCTTTTGGGCGTCGACAAAGACG :  43
Y55  : ATGGGTACTGACTACTACAAGCTTTTGGGCGTCGACAAAGACG :  43
W1   : ATGGGTACTGACTACTACAAGCTTTTGGGCGTCGACAAAGACG :  43
       ATGGGTACTGACTACTACAAGCTTTTGGGCGTCGACAAAGACG

                   *        60         *        80
S606 : CCGACGACAATGCCATTAAGAAAGCTTACAAGAAGATGGCCTT :  86
Y55  : CCGACGACAATGCCATTAAGAAAGCTTACAAGAAGATGGCCTT :  86
W1   : CCGACGACAATGCCATTAAGAAAGCTTACAAGAAGATGGCCTT :  86
       CCGACGACAATGCCATTAAGAAAGCTTACAAGAAGATGGCCTT

         *       100         *       120
S606 : AAAATGGCATCCTGATCGAAACAACGGCTCAGAAGAAGCATCA : 129
Y55  : AAAATGGCATCCTGATCGAAACAACGGCTCAGAAGAAGCATCA : 129
W1   : AAAATGGCATCCTGATCGAAACAACGGCTCAGAAGAAGCATCA : 129
       AAAATGGCATCCTGATCGAAACAACGGCTCAGAAGAAGCATCA

       *       140         *       160         *
S606 : AAAAAGTTCAAAGAGATATCCGAGGCATTCGAAGTTCTGAGCG : 172
Y55  : AAAAAGTTCAAAGAGATATCCGAGGCATTCGAAGTTCTGAGCG : 172
W1   : AAAAAGTTCAAAGAGATATCCGAGGCATTCGAAGTTCTGAGCG : 172
       AAAAAGTTCAAAGAGATATCCGAGGCATTCGAAGTTCTGAGCG

            180         *       200         *
S606 : ACAAACAGAAGCGAACCATATATGATCAGTTCGGTGAAGAGGG : 215
Y55  : ACAAACAGAAGCGAACCATATATGATCAGTTCGGTGAAGAGGG : 215
W1   : ACAAACAGAAGCGAACCATATATGATCAGTTCGGTGAAGAGGG : 215
       ACAAACAGAAGCGAACCATATATGATCAGTTCGGTGAAGAGGG

         220         *       240         *       2
S606 : TCTCAAAGGCGGTGGAGGCCCGCCACCTGGTGCAGGTACCGGT : 258
Y55  : TCTCAAAGGCGGTGGAGGCCCGCCACCTGGTGCAGGTACCGGT : 258
W1   : TCTCAAAGGCGGTGGAGGCCCGCCACCTGGTGCAGGTACCGGT : 258
       TCTCAAAGGCGGTGGAGGCCCGCCACCTGGTGCAGGTACCGGT

       60         *       280         *       300
S606 : GCAGGTCCCAGCGGGTTCTCCGGCTTCAGTGGATTCCCAGGTG : 301
Y55  : GCAGGTCCCAGCGGGTTCTCCGGCTTCAGTGGATTCCCAGGTG : 301
W1   : GCAGGTCCCAGCGGGTTCTCCGGCTTCAGTGGATTCCCAGGTG : 301
       GCAGGTCCCAGCGGGTTCTCCGGCTTCAGTGGATTCCCAGGTG

                *       320         *       340
S606 : GTTCTACATTTAGCTTCTCCAGCTCAGGCCCCTCAGGTTTCAG : 344
Y55  : GTTCTACATTTAGCTTCTCCAGCTCAGGCCCCTCAGGTTTCAG : 344
W1   : GTTCTACATTTAGCTTCTCCAGCTCAGGCCCCTCAGGTTTCAG : 344
       GTTCTACATTTAGCTTCTCCAGCTCAGGCCCCTCAGGTTTCAG
```

```
                 *        360         *        380
S606 : CTCTTCCGGAGGGTCGTTTAATCCATCTGATCCTAATAAGATC : 387
Y55  : CTCTTCCGGAGGGTCGTTTAATCCATCTGATCCTAATAAGATC : 387
W1   : CTCTTCTGGAGGGTCGTTTAATCCATCTGATCCTAATAAGATC : 387
       CTCTTCcGGAGGGTCGTTTAATCCATCTGATCCTAATAAGATC

          *        400         *        420         *
S606 : TTCGAACAAATATTCGGTGTTGGACTCAACTCTGCGTTCGGCA : 430
Y55  : TTCGAACAAATATTCGGTGTTGGACTCAACTCTGCGTTCGGCA : 430
W1   : TTCGAACAAATATTCGGTGTTGGACTCAACTCTGCGTTCGGCA : 430
       TTCGAACAAATATTCGGTGTTGGACTCAACTCTGCGTTCGGCA

              440         *        460         *
S606 : TGGGTGGTATGGGTGGTCGGCCTGGCATGCGAATGTCTGATGC : 473
Y55  : TGGGTGGTATGGGTGGTCGGCCTGGCATGCGAATGTCTGATGC : 473
W1   : TGGGTGGTATGGGTGGTCGGCCTGGCATGCGAATGTCTGATGC : 473
       TGGGTGGTATGGGTGGTCGGCCTGGCATGCGAATGTCTGATGC

           480         *        500         *
S606 : AGACGACGATATGGACGGCTCCTACCCCAGTTTCGGAGGTGGT : 516
Y55  : AGACGACGATATGGACGGCTCCTACCCCAGTTTCGGAGGTGGT : 516
W1   : AGACGACGATATGGACGGCTCCTACCCCAGTTTCGGAGGTGGT : 516
       AGACGACGATATGGACGGCTCCTACCCCAGTTTCGGAGGTGGT

        520         *        540         *        56
S606 : ATACCACGAAGTCGACCATCGCGTCCTGCTCGTTCCAACTCTT : 559
Y55  : ATACCACGAAGTCGACCATCGCGTCCTGCTCGTTCCAACTCTT : 559
W1   : ATACCACGAAGTCGACCATCGCGTCCTGCTCGTTCCAACTCTT : 559
       ATACCACGAAGTCGACCATCGCGTCCTGCTCGTTCCAACTCTT

       0          *        580         *        600
S606 : ATTCTAAACAGTCAGCAGCCCCCTCTGAAATTACCCGTCCATT : 602
Y55  : ATTCTAAACAGTCAGCAGCCCCCTCTGAAATTACCCGTCCATT : 602
W1   : ATTCTAAACAGTCAGCAGCCCCCTCTGAAATTACCCGTCCATT : 602
       ATTCTAAACAGTCAGCAGCCCCCTCTGAAATTACCCGTCCATT

              *        620         *        640
S606 : CAAAGTATCACTGCAGGATCTGTACAACGGGGCTGTCAAGCAT : 645
Y55  : CAAAGTATCACTGCAGGATCTGTACAACGGGGCTGTCAAGCAT : 645
W1   : CAAAGTATCACTGCAGGATCTGTACAACGGGGCTGTCAAGCAT : 645
       CAAAGTATCACTGCAGGATCTGTACAACGGGGCTGTCAAGCAT

           *        660         *        680
S606 : CTCAAAGTCGGGCGAAGACTACTCAACGGTTCGACGGAGGACA : 688
Y55  : CTCAAAGTCGGGCGAAGACTACTCAACGGTTCGACGGAGGACA : 688
W1   : CTCAAAGTCGGGCGAAGACTACTCAACGGTTCGACGGAGGACA : 688
       CTCAAAGTCGGGCGAAGACTACTCAACGGTTCGACGGAGGACA
```

```
               *      700        *      720        *
S606 : AGGTGCTCGACATTCAGGTCCACCCTGGCTGGAAGAGCGGGAC :  731
Y55  : AGGTGCTCGACATTCAGGTCCACCCTGGCTGGAAGAGCGGGAC :  731
W1   : AGGTGCTCGACATTCAGGTCCACCCTGGCTGGAAGAGCGGGAC :  731
       AGGTGCTCGACATTCAGGTCCACCCTGGCTGGAAGAGCGGGAC

            740        *      760        *
S606 : CAAGATTCGCTTTGCGCGCGCAGGCAATGAGCAGGCAAGCGGC :  774
Y55  : CAAGATTCGCTTTGCGCGCGCAGGCAATGAGCAGGCAAGCGGC :  774
W1   : CAAGATTCGCTTTGCGCGCGCAGGCAATGAGCAGGCAAGCGGC :  774
       CAAGATTCGCTTTGCGCGCGCAGGCAATGAGCAGGCAAGCGGC

         780        *      800        *
S606 : GAGGCGCAAGACTTGGTCTTCGTTGTAGAAGAGAAACCGCACG :  817
Y55  : GAGGCGCAAGACTTGGTCTTCGTTGTAGAAGAGAAACCGCACG :  817
W1   : GAGGCGCAAGACTTGGTCTTCGTTGTAGAAGAGAAACCGCACG :  817
       GAGGCGCAAGACTTGGTCTTCGTTGTAGAAGAGAAACCGCACG

       820        *      840        *      860
S606 : ATACCTTCAAGCGAGAAGGGAATGACCTTATCTGTAACGTTTC :  860
Y55  : ATACCTTCAAGCGAGAAGGGAATGACCTTATCTGTAACGTTTC :  860
W1   : ATACCTTCAAGCGAGAAGGGAATGACCTTATCTGTAACGTTTC :  860
       ATACCTTCAAGCGAGAAGGGAATGACCTTATCTGTAACGTTTC

                *      880        *      900
S606 : AATACCCCTTCTGGAGGCCTTGACGCACGAAGGTGGCAAGAAG :  903
Y55  : AATACCCCTTCTGGAGGCCTTGACGCACGAAGGTGGCAAGAAA :  903
W1   : AATACCCCTTCTGGAAGCCTTGACGCACGAAGGTGGCAAGAAG :  903
       AATACCCCTTCTGGAgGCCTTGACGCACGAAGGTGGCAAGAAg

             *      920        *      940
S606 : CAAGTGGAATCGCTGGACGGCGGAAGATACAAGTGGATTTAC :  946
Y55  : CAAGTGGAATCGCTGGACGGCGGAAGATACAAGTGGATTTAC :  946
W1   : CAAGTGGAATCGCTGGACGGCGGAAGATACAAGTGGATTTAC :  946
       CAAGTGGAATCGCTGGACGGCGGAAGATACAAGTGGATTTAC

          *      960        *      980
S606 : CTGCTGGTGTTATCAAACCCGGGCAGGAGACAACTGTTCATGG :  989
Y55  : CTGCTGGTGTTATCAAACCCGGGCAGGAGACAACTGTTCATGG :  989
W1   : CTGCTGGTGTTATCAAACCCGGGCAGGAGACAACTGTTCATGG :  989
       CTGCTGGTGTTATCAAACCCGGGCAGGAGACAACTGTTCATGG

       *     1000        *     1020        *
S606 : AGAGGGTATGCCTATCAGGAAAGACGGCATGGTGAAGAAAAAG : 1032
Y55  : AGAGGGTATGCCTATCAGGAAAGACGGCATGGTGAAGAAAAAG : 1032
W1   : AGAGGGTATGCCTATCAGGAAAGACGGCATGGTGAAGAAAAAG : 1032
       AGAGGGTATGCCTATCAGGAAAGACGGCATGGTGAAGAAAAAG
```

```
          1040         *        1060         *
S606 : GGTGATTTGATTGTGAAATGGAATGTCGTCTTCCCAGATCGAT : 1075
Y55  : GGTGATCTGATTGTGAAATGGAATGTCGTCTTCCCAGATCGAT : 1075
W1   : GGTGATCTGATTGTGAAATGGAATGTCGTCTTCCCAGATCGAT : 1075
       GGTGATcTGATTGTGAAATGGAATGTCGTCTTCCCAGATCGAT

        1080         *        1100         *
S606 : TAACATCGTCGCAGAAGGCGGGATTGAAAAAGGTCCTTTCATG : 1118
Y55  : TAACATCGTCGCAGAAGGCGGGATTGAAAAAGGTCCTTTCATG : 1118
W1   : TAACATCGTCGCAGAAGGCGGGATTGAAAAAGGTCCTTTCATG : 1118
       TAACATCGTCGCAGAAGGCGGGATTGAAAAAGGTCCTTTCATG


S606 : A : 1119
Y55  : A : 1119
W1   : A : 1119
       A
```

FigureS2 Self-activation assay.

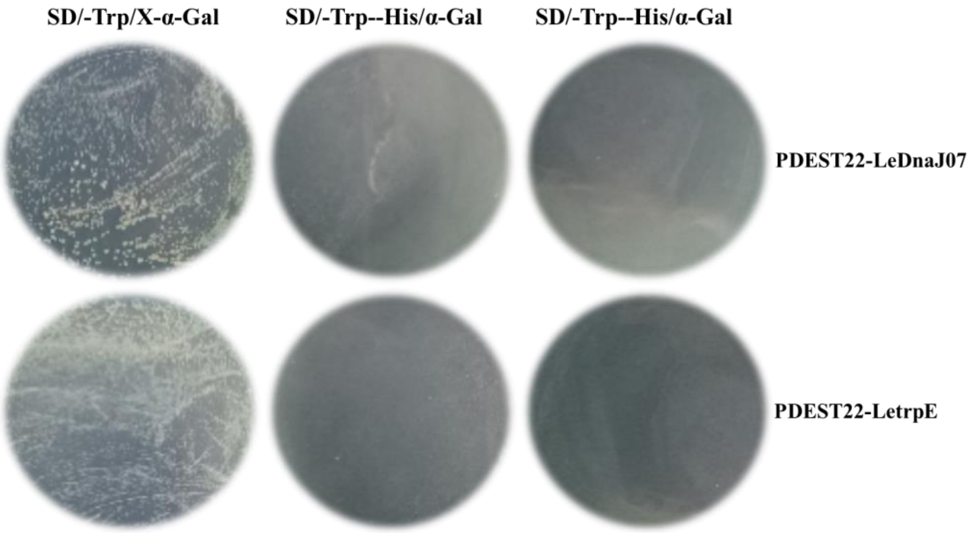

Table S1 Primers used in this work.

Primers	sequence
qLeDnaJ-F	AGAGAAACCGCACGATACCT
qLeDnaJ-R	ACCATGCCGTCTTTCCTGAT
qLeactin-F	GGAGAAGATTTGGCATCACACA
qLeactin-R	GAAGAGCGAAACCCTCGTAGA
OLeactin-F	ccacctcaaacttcggaattcAGGGACTAGAACAAAAGGGTATGTG
OLeactin-R	tgctcaccatCTACAGCAGTCTTAGCATCGAGTATATAA
Omecherry-F	actgctgtagATGGTGAGCAAGGGCGAGG
Omecherry-R	cagtacccatCTTGTACAGCTCGTCCATGCC
OLeDnaJ-F	gctgtacaagATGGGTACTGACTACTACAAGCTTTTG
OLeDnaJ-R	tctagaggatccccgggtaccTCATGAAAGGACCTTTTTCAATCC

Note: For the primers constructing the vector, lowercases indicate the homologous arm, and capitals indicates the primers of the gene fragments.
